# Chronic Psychological Stress Was Not Ameliorated by Omega-3 Eicosapentaenoic Acid (EPA)

**DOI:** 10.3389/fphar.2017.00551

**Published:** 2017-10-31

**Authors:** Joanne Bradbury, Stephen P. Myers, Barbara Meyer, Lyndon Brooks, Jonathan Peake, Andrew J. Sinclair, Con Stough

**Affiliations:** ^1^School of Health and Human Sciences, Southern Cross University, Gold Coast, QLD, Australia; ^2^NatMed-Research, Division of Research, Southern Cross University, Lismore, NSW, Australia; ^3^Faculty of Science Medicine and Health, Lipid Research Centre, School of Medicine, University of Wollongong, Wollongong, NSW, Australia; ^4^Division of Research, Southern Cross University, Lismore, NSW, Australia; ^5^School of Biomedical Sciences and Institute of Health and Biomedical Innovation, Queensland University of Technology, Brisbane, QLD, Australia; ^6^Faculty of Health, Office of Faculty of Health, Deakin University, Melbourne, VIC, Australia; ^7^Centre for Human Psychopharmacology, Swinburne University of Technology, Melbourne, VIC, Australia

**Keywords:** psychological stress, chronic stress, docosahexaenoic acid (DHA), omega-3 fatty acids, eicosapentaenoic acid (EPA), fish oil, proinflammatory cytokines, coping

## Abstract

**Background:** Chronic psychological stress and mental health disorders are endemic in Western culture where population dietary insufficiencies of omega-3 fatty acids (n-3FA) from seafood have been observed.

**Objective:** This study was designed to test for a causal relationship between one of the most active components of fish oil, eicosapentaenoic acid (EPA), and chronic psychological stress.

**Method:** A randomized double-blind, placebo-controlled clinical trial with parallel-assignment to two groups was designed (Trial Id: ACTRN12610000404022). The interventions were four EPA-rich fish oil capsules per day, delivering 2.2 g/d EPA (and 0.44 g/d DHA), or identical placebo (low-phenolic olive oil capsules with 5% fish oil to aid blinding). The primary outcome was the between-group difference on the Perceived Stress Scale (PSS-10) after 12 weeks supplementation. An a priori power analysis determined that group sizes of 43 would provide 80% power to detect a significant between-group difference of 12.5%, at α = 0.05. Ninety community members (64 females, 26 males) reporting chronic work stress were recruited via public advertising in northern NSW, Australia.

**Results:** At baseline the omega-3 index (EPA + DHA as % to total fatty acids in red blood cell membranes) was 5.2% in both groups (*SD* = 1.6% control group; 1.8% active group). After supplementation this remained stable at 5.3% (*SD* = 1.6%) for the control group but increased to 8.9% (*SD* = 1.5%) for the active group, demonstrating successful incorporation of EPA into cells. Intention-to-treat (ITT) analysis found no significant between-group differences in PSS outcome scores post-intervention (*b* = 1.21, *p* = 0.30) after adjusting for sex (*b* = 2.36, *p* = 0.079), baseline PSS (*b* = 0.42, *p* = 0.001) and baseline logEPA [*b* = 1.41, *p* = 0.185; *F*_(3, 86)_ = 8.47, *p* < 0.01, *n* = 89, R-square = 0.243].

**Discussion:** Treatment increased cell membrane EPA but, contrary to the hypothesis, there was no effect on perceived stress. Limitations included an imbalance of gender in groups after randomization (68% of the males were in the placebo group). While we found no significant interaction between sex and group on the outcome after adjusting for baseline PSS, larger studies with groups stratified for gender may be required to further confirm these findings.

**Conclusion:** This study demonstrated that 2. 2 g/day of EPA for 12 weeks did not reduce chronic psychological stress.

## Introduction

Chronic psychological stress is prevalent in Australia and all Western countries. Exposure to chronic psychological stress has been linked to an increased risk of diseases. It doubles risk of cardiovascular disease, depression and anxiety (LaMontagne et al., 2006) and accelerates markers of aging, such as decreased telomerase activity (Deng et al., 2016). Dietary (e.g., supplementation with omega-3 fatty acids and/or antioxidants) and lifestyle (e.g., physical exercise, mindfulness practices) factors are increasingly recognized as important mediators of resilience to the damaging effects of chronic stress (Buchholz, 2015).

Psychological stress is mediated through limbic centers in the brain, and involves a dynamic interplay between the amygdala (brain region involved with fear/apprehension) and the hippocampus (brain region involved with learning and experience), with an executive regulation role for the prefrontal cortex (cognitive appraisal). The biological stress response is activated if the psychological appraisal of the threat results in the perception that the demands will exceed the ability to cope with those demands. Stress appraisal measurement is based on the subjective evaluation of the stressor (primary appraisal) and available coping resources (secondary appraisal; Lazarus and Folkman, 1984). The net balance of these appraisals determines the extent of physiological stress activation, if any (Rohrmann et al., 1999; Ennis et al., 2001).

Physiological stress activation is mediated by the hypothalamus-pituitary-adrenal (HPA) axis. The immediate, acute response involves hypothalamic activation of sympathetic nervous system and its activation of inflammatory pathways in order to prepare the body for breaches to the skin and contain blood loss and infection (Dhabhar, 1998). In acute situations this is a survival mechanism, but long term this creates a prothrombotic, proinflammatory physiology that itself becomes stressful to the body if perpetuated (Gold, 2015). Cytokines are messenger molecules that are produced by white blood cells during stress. Overproduction of the proinflammatory cytokines during stress stimulates the hypothalamus to continue activation of the stress response (Elenkov and Chrousos, 2002). Whether this is a healthy or dysfunctional effect depends upon the situation. However, proinflammatory cytokines have been demonstrated to prevent the HPA axis from efficiently returning to homeostasis, leading to ongoing activation (Leonard, 2005). HPA axis dysregulation has been associated with aging (Seeman and Robbins, 1994) and stress- and mood-related disorders, such as depression (Leonard and Song, 1996; Anisman and Merali, 2003).

Cortisol is the end product of HPA axis activation. It is an anti-inflammatory fat-soluble steroid hormone that is released by the adrenal gland during intense or prolonged stress. It is adaptogenic, as it helps the organism to sustain its response to ongoing stress by modulating the impact of the proinflammatory stress mediators. However, sustained high levels of cortisol causes neuronal damage in the hippocampus (Frodl and O'Keane, 2013), a brain region critical for higher order stress regulation. Dehydroepiandrosterone (DHEA) is another steroid hormone that is released alongside cortisol during stress. Its action is antagonistic to cortisol and is known as the anti-stress hormone (Hu et al., 2000; Bauer et al., 2013). The ratio between these two hormones has been suggested as a marker of resilience to stress (Yehuda et al., 2014).

Dietary omega-3 fatty acids (n-3FAs) have similar properties to cortisol and also have adaptogenic effects (Hamazaki et al., 2000; Bradbury et al., 2004); they are fat-soluble, anti-inflammatory in nature and are known regulators of proinflammatory cytokine production (Calder, 2006). *In vitro* studies have shown that the n-3FAs eicosapentaenoic acid (EPA) and docosahexaenoic acid (DHA) actively suppress IL-6 production by human endothelial cells (De Caterina et al., 1994; Khalfoun et al., 1997) and the production of IL-1, IL-6, and TNF by human lymphocytes (Purasiri et al., 1997). Studies have also established that dietary supplementation with EPA and DHA suppresses IL-1, IL-6, and TNF production by monocytes in rats (Foitzik et al., 2002). The addition of the omega-3 rich flaxseed oil to domestic food products was shown to reduce proinflammatory cytokine production in humans (James et al., 2000). A significantly higher proinflammatory response to stress during exams was demonstrated in medical students who had lower baseline levels of n-3FAs (Maes et al., 2000).

In addition to their anti-inflammatory effects in the immune system, n-3FAs seem to have protective effects on stress-related brain regions. In animal studies EPA was demonstrated to be neuroprotective in the hippocampus against stress-induced cell death in rats. The mechanism appeared to involved upregulation of IL-10 (Lynch et al., 2004). To mimic a stress response, lipopolysaccharide (LPS) is used to induce proinflammatory IL-1β levels in the hippocampus, through upregulation in the expression of the IL-1 receptor Type I protein (IL-1RI). Increased IL-1RI activates a cascade of pathways resulting in neuronal cell death and consequential interference in long-term potentiation (involved with the laying down of new memories). However, these LPS-induced changes were prevented with the administration of the regulatory cytokine IL-10 (Lynch et al., 2004). This suggests the potential for an active role of stress regulation by IL-10 at the hippocampal level.

EPA was shown to enhance hippocampal IL-10 levels, which were inversely correlated with IL-1β (Lynch et al., 2003). In a study involving whole body exposure to irradiation in rats, IL1β levels were increased in the hippocampus, which activated the cascade of events leading to cell death and interference of long-term potentiation. However, rats that were given EPA prior to exposure were completely spared the radiation-induced cell death in the hippocampus, and did not exhibit interruptions in long-term potentiation. This suggests the potential for an active role for EPA in stress regulation.

Most Australians are failing to meet the minimum intake requirement for the long chain omega-3 fatty acids. The National Health and Medical Research Centre (NHMRC) established Suggested Dietary Targets (SDT) for the long chain omega-3 fatty acids at 610 mg/d for men and 430 mg/d for women to reduce chronic disease risk (NHMRC 2006). In 2006, the Australian median daily intake was only 121 mg/d (Meyer et al., 2003; Howe et al., 2006; Meyer, 2011). This indicates that most Australians are consuming about one quarter of the recommended daily requirements of these essential nutrients. This remained unchanged when 2011-2012 Australian data was analyzed (Meyer, 2016). This is concerning given that Hibbeln (Hibbeln, 1998) demonstrated that fish consumption per capita was inversely correlated with the annual prevalence of major depression per country.

If n-3FAs regulate cytokine production during chronic stress, then intervention with dietary n-3FAs may reduce stress-related susceptibility to disease. To test this theory, this study aimed to demonstrate whether dietary supplementation with an EPA-rich fish oil can alleviate perceived stress in people with chronic stress compared with a placebo. Chronic work stress was used as a relatively stable model of stress in human participants. As it was postulated that EPA is the n-3FA most likely to have an impact on inflammatory mediators, an EPA-rich fish oil was chosen as the predominant fish oil component for the active intervention. The hypothesis was that EPA would be better than placebo in reducing perceived stress.

## Materials and methods

### Study design

**Figure 1:**
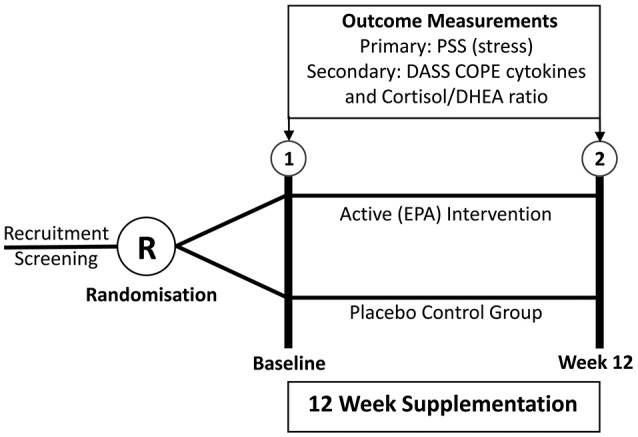
Design of RCT.

The study was designed as a randomized, placebo-controlled, double-blind, parallel-assignment clinical trial. The two intervention groups for comparison were the active fish oil (FO) group and the placebo control group, as shown in Figure 1. The study design was registered prospectively at the Australian Clinical Trials Register (Trial Id: ACTRN12610000404022).

### Study protocol

Participants were required to attend the sampling clinic on two occasions, baseline and post-intervention, for about an hour. All clinics were conducted between 7 and 10 a.m. Participants were required to fast from all food and beverage (except for water), and to refrain from over-the-counter medications and heavy physical exercise for the 12 h prior to the clinic. Questionnaires were administered, and blood and saliva samples were collected at baseline, then again after 12 weeks of supplementation. Trained clinicians conducted a clinical interview and full physical examination of participants on each occasion; including height, weight, body mass index, waist circumference, blood pressure, and heart rate.

In addition to the sampling clinics, participants were required to attend a “bottle exchange” clinic once per month to return the current bottle with whatever capsules remained and to collect a new bottle containing a month's supply.

Outside the clinics every 2 weeks during the study the PSS questionnaire was emailed to participants to complete and return within 72 h to monitor the stress levels during the study.

### Outcome measures

The primary outcome measure was the difference between the fish oil group and placebo group in mean changes over time on the ten-item Perceived Stress Scale (PSS). The secondary outcome measures were the differences between the fish oil group and placebo group in mean changes over time in (i) plasma concentrations of IL-1β, IL-6, TNF-α, and IL-10 (ii) salivary cortisol/DHEA ratio, (iii) Depression, Anxiety, Stress Scale (DASS), (iv) Occupational Stress Inventory (OSI) Strain and Resources subscales, (v) COPE Inventory, and (vi) Copenhagen Burnout Inventory.

### Study population

This study was carried out in accordance with the Australian Government's National Statement on Ethical Conduct in Human Research (2007)—Updated May 2015. The protocol was approved by the Southern Cross University Human Research Ethics Committee. Paid advertisements and media coverage in regional papers and radio stations advertised that the study was particularly targeting people with moderate to high levels of work stress. Volunteers who met the study criteria were randomized into one of two groups.

### Randomization, concealment, and blinding

An independent staff member not associated with the current study or funding bodies, conducted the randomization and coding of the supplement bottles. A computer program was used to generate the randomization schedule. The coding was kept in sealed, opaque envelopes in a locked filing cabinet by this staff member until the completion of the trial. The participants, data collection clinicians, researchers, laboratory and statistical analyses were all blinded to the allocation to groups throughout the study. Blinding was aided by the addition of 5% fish oil to the placebo capsules by the manufacturer. The placebo capsules were identical to the EPA capsules in size, shape, color, smell and taste.

### Sample size determination

In our previous pilot randomized controlled intervention study (Bradbury et al., 2004), our sample of 30 participants with chronic work stress obtained a mean baseline score of PSS = 24 and a 26.7% [6.4 (±5.8)] mean reduction from baseline to completion with treatment with fish oil (FO) and a 12.5% [3.0 (±4.8)] mean reduction with placebo. A power analysis based on these parameters determined that equal group sizes of *n* = 43 would provide at least 80% power to detect a significant difference between groups, assuming a reduction of 25% [6.0 (±6.0)] for the fish oil group and a reduction of 12.5% [3.0 (±5.0)] for the placebo, controlling alpha at 0.05 for a one-tailed test. In case we observed a 30% drop out rate, as occurred in the pilot study, we aimed to recruit 55 subjects in each of the two groups to allow for a fully powered per-protocol hypothesis test, secondary to the intention-to-treat (ITT) analysis. Hence, it was anticipated that *n* = 110 would provide the study with the best chance of finding a significant difference between the groups if one exists in the population of people with chronic psychological stress.

### Study criteria

Participants were included in the study if they reported experiencing chronic work stress and if they obtained moderate to high stress scores (PSS ≥ 17); they reported having not taken a course of fish oil within the past 3 months, and/or that they consumed less than two non-fried fish meals per week on average; and that they were willing to refrain from non-steroidal anti-inflammatory drugs, aspirin, and other complementary medicine supplements for the trial duration. Participants were excluded from the study if they were taking immunosuppressive medications; or had any systemic inflammatory disorder; a current infection; unexplained weight loss; malignancy; or any significant disease or disorder or any condition that, in the opinion of the investigators, might interfere with the study objectives.

### Study medication

Eicosapentaenoic acid (EPA) from fish oil constituted the active intervention. Each capsule contained 1,000 mg fish oil, comprising 660 mg marine triglycerides as EPA 550 mg and DHA 110 mg. Four capsules per day delivered 2.2 g EPA, and 0.44 g DHA. This equated to a total of 2.64 g per day of the long chain omega-3 fatty acids. The placebo capsules, contained 950 mg low-phenolic olive oil, consisting predominately of monounsaturated fatty acids, and 50 mg fish oil (5%) to assist with blinding. The intervention involved participants taking four capsules per day, two with breakfast and two with dinner. All bottles with any remaining supplements were collected to determine how many capsules remained as a measure of compliance.

### Measurement instruments

#### PSS-10

The PSS (Cohen et al., 1983) is a validated and reliable subjective measure of the amount of psychological stress an individual perceives. The questions ask respondents about thoughts and feelings they have experienced during the last month. Items are answered in a Likert fashion from “never” = 0 to “very often” = 4. Scoring of the PSS involves summing across the 10 items, resulting in a single score in the range from 0 to 40. Higher scores reflects higher perceived stress.

#### Membrane fatty acids

Venous samples were collected in 4 mL EDTA tubes, which were gently inverted at the time of collection. Upon arrival at the lab (Centre for Phytochemistry & Pharmacology at Southern Cross University), the tubes were centrifuged for 5 min at 2,000 g (3,057 rpm) at room temperature (25°C). The plasma was separated from the packed red cells at the bottom of the tube, leaving ~2 ml of plasma and 2 ml of red blood cells (RBC). The plasma and the buffy coat were transferred by pipette out of the tubes. The packed red cells at the bottom of the tube were transferred into Eppendorf tubes and stored at −80°C. At the end of the data collection period, they were transported as a batch on dry ice to the University of Wollongong for fatty acid analysis. RBC Fatty Acid Analysis (FAA) was conducted using the protocol previously published by Lepage and Roy (1986). Through comparison with known fatty acid standards, individual fatty acids were identified. The standards used were from Supelco FAME mix C4-C24 analytical standard (Product #18919-1 AMP, Sigma-Aldrich, Castel Hill, Australia).

#### Proinflammatory cytokines

EDTA tubes were used to collect 5 ml of plasma for the cytokines, while 5 ml blood was collected in serum separation tubes for the analysis of serum C-reactive protein concentration. Samples were transported directly to the Centre for Phytochemistry & Pharmacology at Southern Cross University, where the plasma was centrifuged for 10 min at 4°C at 2,500 rpm. Serum was similarly centrifuged after it had been separated for 10 min at room temperature. The plasma and serum were divided into 500 ml aliquots, stored in Eppendorf tubes and frozen at −80°C. At the end of data collection, samples were transferred in one batch on dry ice to the University of Queensland. Plasma samples were analyzed in single using ELISA kits for IL-1β, IL-6, and TNF-α (Quantikine HS®, RnD Systems, Minneapolis, MN) and IL-10 (Quantikine®, RnD Systems) according to the manufacturer's instructions. Serum samples were analyzed in single for high-sensitivity C-reactive protein using a commercial kit (Kamiya Biomedical, Seattle, WA) according to the manufacturer's instructions and an automated spectrophotometer (Cobas MIRA, Roche Diagnostics, Basel, Switzerland).

#### Salivary cortisol/DHEA ratio

During the data collection clinics and prior to blood analysis, saliva was collected in 1.5 ml conical microtubes (Quality Scientific Plastics, Petaluma, California), which were transferred in a transportable insulated box from the clinic to the laboratory to be stored in a −20°C freezer until the samples from the complete study had been collected. They were then transferred as a batch to the −80°C laboratory freezer where they were stored until they were thawed for analysis. Saliva was collected using a small plastic 6 cm straw, according to the “passive drool” collection. Briefly the participants were asked to allow saliva to run into the straw provided for collection into the tube, which was held directly under the mouth. Immediately prior to collection, participants were asked to rinse out their mouth with water and then to sit comfortably and wait for saliva that was naturally accumulating in the mouth to drip through the straw into the tube. Salivary cortisol and DHEA were, respectively, analyzed using the manufacturer's instructions with standard salivary ELISA kits (IBL, Australia).

#### The depression, anxiety, stress scale (DASS-21)

The brief (21-item) DASS (Lovibond and Lovibond, 1995) is a validated and reliable subjective measure of three constructs; stress, anxiety and depression. There are seven questions pertaining to each construct. The items for each construct are summed, giving a total score for each subscale. Subscale scores range between 0 and 21.

#### The occupational stress inventory- revised (OSI-R)

The OSI-R (Osipow, 1981) is a multidimensional measure of occupational stress. It is a self-report instrument involving three questionnaires. There are six subscales in the Occupational Roles Questionnaire (ORQ), four subscales in the Psychological Strain Questionnaire (PSQ) and four subscales in the Personal Resources Questionnaire (PRQ). Each scale consists of several subscales. Each subscale consists of 10 items. Each item is answered in a five point Likert response ranging from “rarely or never” to “most of the time.” Reliability Chronbach alpha coefficients were demonstrated to range from 0.7 to 0.89 among the subscales, and 0.88 for the ORQ, 0.93 for the PSQ and 0.89 for the PRQ. Only the Strain and Resources subscales were used in this study.

#### The COPE inventory

The COPE (Carver et al., 1989) is a reliable and validated measure of coping styles. It consists of 15 subscales, addressing both positive (such as use of humor) and negative (such as denial) coping mechanisms. Items are measured on a four-point Likert scale from 1 = “I haven't been doing this at all” to 4 = “I have been doing this a lot.” The scores on each subscale range between 4 and 16. The COPE is a most widely used measure of coping due to its derivation from theory and its standardized scoring procedure.

#### Burnout

Burnout is a prolonged physical and psychological exhaustion, that has been associated with chronic work stress (Maslach et al., 2001). In order to control for this as a potential covariate, the Copenhagen Burnout Inventory (Kristensen et al., 2005) was included.

### Statistical analysis

Red blood cell membrane EPA (mEPA) was assessed at baseline in both groups as the most significant potential covariate. Pre-treatment levels would be included in the statistical model should the groups differ at baseline. The success of the random assignment to groups would be further tested in terms of age, gender and other potential covariates, which would also be included were they to differ between groups at baseline. ITT analysis was used for the primary analysis, incorporating a general linear model to adjust for baseline stress levels and covariates, where required. Probability levels ≤ 0.05 was considered significant for the primary analysis, secondary analyses would be treated as exploratory. Statistical analyses were conducted using Stata v13.

## Results

### Participants

**Figure 2:**
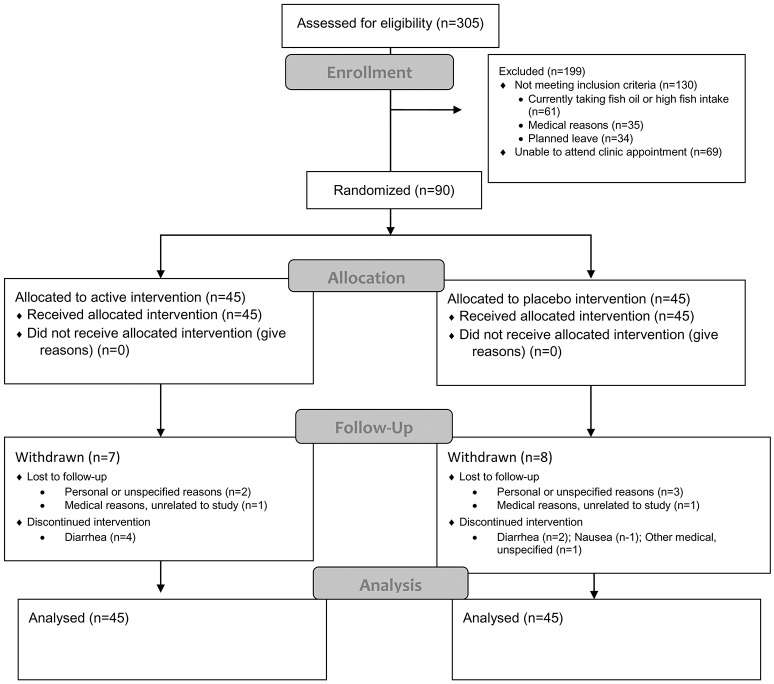
Flow of participants through the study.

Ninety participants (*n* = 90, 64 female and 26 male) were randomized. Figure 2 shows the flow of participants through the study. During the 3 months of the supplementation period, 15 participants (2 male and 13 female) withdrew for various personal or medical reasons. Most were unrelated to the intervention, but there were issues of compliance with the protocol, particularly of continuing to take the fish oil supplement and abstain from taking other supplements. One withdrawal was reportedly associated with an adverse reaction of mild abdominal bloating attributed to the intervention. However, no withdrawal-retest was conducted to demonstrate whether the intervention caused the symptom.

### Baseline characteristics

Baseline demographic and clinical characteristics were fairly evenly distributed between the groups as shown in Table 1. However, there was a lower proportion of males allocated to the active (fish oil) group compared with the placebo control group (68 vs. 32%). In addition, a higher proportion of people experiencing financial stress (69 vs. 31%) and a significant trauma in the 6 months prior to the study were allocated to the active fish oil group (70 vs. 30%).

**Table 1:** Baseline demographic and clinical characteristics for the two groups goes here.

		**Active treatment group (*N* = 45) Mean (Standard Deviation)**	**Placebo control group (*N* = 45) Mean (Standard Deviation)**
Age (years)		43.24(9.52)	45.38(9.17)
Sex	Female	37(56.9%)	28(43.1%)
	Male	8(32.0%)	17(68.0%)
Body Mass Index		25.3(4.9)	26.4(5.0)
Waist circumference (cm)		65.8(33.5)	62.1(38.0)
Heart rate (beats per min)		67.3(11.2)	67.1(11.2)
Blood pressure (mm Hg)	Systolic	126.4(16.3)	126.9(15.3)
	Diastolic	78.8(11.3)	78.6(11.4)
		**Number (percentage)**	**Number (percentage)**
Primary carer status	Child, elderly, disable person	21(53.8)	18(46.2)
Relationship status	Single	7(63.6)	4(36.4)
	Widowed/divorced	30(57.1)	22(42.9)
	Married	31(45.6)	37(54.4)
	Recent loss	2(66.7)	1(33.3)
Ethnicity	White Caucasian	43(48.9)	45(51.1)
	Aboriginal/Torres St Is	1(100)	0(0)
	Other	1(100)	0(0)
Occupation status	Manager	9(40.9)	13(59.1)
	Professional	17(50)	17(50)
	Associate professional	12(75)	4(25)
	Trades	0(0)	3(100)
	Advanced clerical/service	3(42.9)	4(57.1)
	Sales and service	0(0)	3(100)
	Elementary clerical, sales, service	2(100)	0(0)
	Labourer	1(50)	1(50)
Financial Stress	Unable to raise $2,000	18(69.2)	8(30.8)
Significant trauma	Within 6 months	16(69.6)	7(30.4)

### Descriptive statistics

The group means and standard deviations at baseline (pre) and at post-intervention (post) for the serum fatty acids are given in Table 2. There were no significant differences between the groups at baseline for any of the fatty acids, while there were significant differences in the fatty acids at Time 2. The main differences were a dramatic increase in the EPA content of RBC membranes, in both absolute levels (μg/ml) and as a percentage of total fatty acids(%), with a corresponding decrease in arachidonic acid levels for the fish oil group compared with controls. The pre and post group means for the secondary outcomes are provided in Table 3.

**Table 2:** Group means and standard deviations at baseline (pre) and at post-intervention (post) for the membrane and serum fatty acids.

**Membrane RBC fatty acids**	**Baseline (pre-intervention,** ***n*** = **89)**		**Time 2 (post-intervention,** ***n*** = **74)**		**Between-group difference atpost-intervention**		
	**Fish oil group(*N* = 45)**	**Placebo(*N* = 45)**	**Fish oil group(*N* = 38)**	**Placebo(*N* = 36)**	**Diff**.	***t***	***p***
**ABSOLUTE LEVELS (μg/ml)**							
Arachidonic acid	59.0(26.3)	56.5(27.5)	36.4(10.2)	45.0(12.4)	−8.7	3.29	0.002^*^
EPA	6.1(10.2)	4.3(4.9)	13.4(7.5)	3.8(3.3)	9.6	−7.06	<0.001^*^
DHA	19.1(9.1)	19.5(11.1)	16.9(1.4)	15.0(5.4)	1.9	−1.59	0.117
**PERCENTAGE (%) OF TOTAL FATS**							
Arachidonic acid	13.3(1.4)	13.2(1.2)	10.9(1.1)	13.2(1.2)	−2.3	8.69	<0.001^*^
EPA	1.2(1.4)	1.0(1.0)	4.1(1.2)	1.2(1.3)	2.9	−10.05	<0.001^*^
DHA	4.0(0.9)	4.2(1.4)	4.8(0.7)	4.1(1.0)	0.7	−3.51	0.001^*^
EPA + DHA (%)	5.2(1.6)	5.2(1.8)	8.9(1.5)	5.3(1.6)	3.6	−10.35	<0.001^*^
AA:EPA (%)	16.5(8.6)	16.8(6.7)	3.0(1.2)	16.0(6.9)	−13.0	11.46	<0.001^*^
EPA:AA (%)	0.10(0.16)	0.08(0.10)	0.38(0.15)	0.10(0.11)	3.00	9.10	<0.001^*^

Diff, mean (Active group)—mean (Placebo group) at post-intervention; EPA + DHA is known as the “omega-3 index.”

**Table 3:** Baseline and Post intervention group means and standard deviations for secondary outcomes.

	**Baseline (Pre-intervention)**		**Time 2 (Post-intervention)**	
**Salivary biomarkers (nmol/L)**	**Fish oil group (*****N*** **= 40)**	**Placebo (*****N*** **= 39)**	**Fish oil group (*****N*** **= 38)**	**Placebo (*****N*** **= 36)**
Cortisol	12.65(7.29)	13.62(8.93)	15.95(8.11)	13.40(7.22)
DHEA	0.97(0.65)	1.24(0.94)	1.22(1.03)	1.48(1.86)
Cortisol:DHEA	16.98(11.48)	14.43(10.37)	19.12(13.39)	15.57(11.87)
**Serum inflammatory markers**	**Fish oil group (*****N*** **= 45)**	**Placebo (*****N*** **= 44)**	**Fish oil group (*****N*** **= 36)**	**Placebo (*****N*** **= 36)**
TNF pg/ml	0.97(0.65)	0.93(0.48)	1.23(1.0)	1.07(0.67)
IL-6 pg/ml	0.90(0.81)	0.95(0.74)	0.91(0.69)	1.00(0.97)
IL-10 pg/ml	2.6(1.150)	2.12(0.99)	2.15(1.22)	2.01(0.93)
CRP mg/l	1.33(1.49)	1.19(1.48)	1.18(1.38)	0.84(0.96)
**Psychological self-report scales**	**Fish oil group (*****N*** **= 40)**	**Placebo (*****N*** **= 44)**	**Fish oil group (*****N*** **= 29)**	**Placebo (*****N*** **= 29)**
OSI Roles	158.2(26.7)	155.8(27.8)	149.9(27.1)	145.8(21.5)
OSI Strain	109.8(21.4)	107.8(23.2)	92.4(17.2)	90.5(21.2)
OSI Resources	110.3(20.4)	111.6(19.5)	114.9(19.1)	116.9(18.1)
DASS Stress	19.3(9.6)	19.5(9.0)	14.7(7.8)	15.3(8.4)
DASS Anxiety	9.7(8.4)	9.32(9.5)	6.2(6.3)	7.0(8.6)
DASS Depression	15.6(10.8)	12.7(10.8)	10.9(8.9)	12.2(10.7)
COPE Active	10.2(2.5)	10.5(1.5)	10.2(2.4)	10.7(2.6)
COPE Denial	6.0(2.0)	6.2(2.0)	5.3(2.1)	5.9(2.2)
COPE Disengagement	7.4(2.3)	6.2(2.0)	6.6(1.9)	6.2(1.9)
Burnout	58.0(16.7)	58.0(16.8)	56.2(19.5)	50.0(20.0)

### Primary outcome

**Figure 3:**
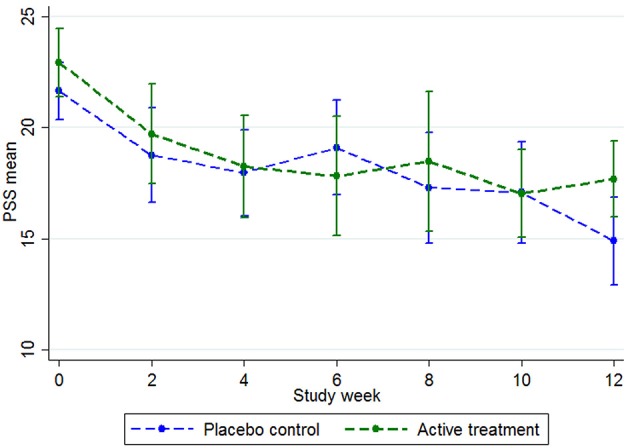
PSS group means during the trial, with 95% confidence intervals. Baseline *n* = 90, week 2 *n* = 65, week 4 *n* = 70, week 6 *n* = 63, week 8 *n* = 52, week 10 *n* = 62, week 12 *n* = 75.

An ITT analysis was conducted for the difference between groups at the post-intervention point for the primary outcome, the PSS. As the PSS had been administered every 2 weeks during the study (see Figure 3), the last observed value for the PSS was brought forward and used in the post-intervention (Time 2) analysis (*n* = 90). That is, there were *n* = 75 participants in week 12, but by using the last known PSS for the participants who withdrew, the Time 2 analysis was conducted on all participants who were randomized (*n* = 90).

At baseline, the total scores on the PSS-10 were mean (*M* = 21.68; *SD* = 4.43) for the placebo group and *M* = 22.93 (*SD* = 5.18) for the active group (Figure 3). Both groups steadily declined in stress levels, with the placebo group declining more than the active group at the week 12 mark (Figure 3). When the last values were bought forward, the post-intervention stress scores were *M* = 16.07 (*SD* = 6.28) for the placebo control and *M* = 18.36 (*SD* = 5.39) for the active group, which was not a significant between-group difference (*t* = 1.86, *p* = 0.07).

Sex, financial stress and significant trauma in the 6 months prior to the study were checked at baseline. Sex was found to be a significant cofactor, with the mean PSS for males (*M* = 20.56, *SD* = 5.69) lower than for females (*M* = 22.98, *SD* = 4.32; *t* = −2.18, *p* = 0.03) at baseline and post-intervention males (*M* = 14.32, *SD* = 5.28) vs. females (*M* = 18.32, *SD* = 5.83; *t* = −2.99, *p* = 0.004). Because sex was not balanced after randomization, an interaction term for sex^*^group was modeled, but was non-significant and therefore not included in the final model.

There was also a potential imbalance in the variance in absolute levels of membrane EPA (μg/mL). At baseline, the fish oil group had a mean EPA of 6.1 μg/mL (*SD* = 10.2 μg/mL) where the control group had 4.3 (4.9) μg/mL (Table 2). The distribution was highly positively skewed in both groups. The natural log of EPA (lnEPA) as a percentage to total fatty acids was taken as the best marker for membrane EPA, with notable improvement in its distribution.

While there were no between-group differences in lnEPA, there was a trend toward sex differences, with females trending higher than males (mean difference 0.21, *p* = 0.099). The interaction with sex and EPA was modeled with the outcome but found to be non-significant when two influential cases were removed. Baseline EPA was not a significant covariate in the model but was nevertheless retained as it was part of the a priori study design to control for subtle differences at baseline.

In the final model, ITT analysis found no significant between-group differences in PSS outcome scores post-intervention (*b* = 1.21, *p* = 0.30) after adjusting for sex (*b* = 2.36, *p* = 0.079), baseline PSS (*b* = 0.42, *p* = 0.001) and baseline lnEPA [*b* = 1.41, *p* = 0.185; *F*_(3, 86)_ = 8.47, *p* < 0.01, *n* = 89, R-square = 0.243] (Table 4).

**Table 4:** Coefficient table for the effect of treatment group on PSS at post-intervention (*n* = 90).

**PSS-post**	**Coef**.	**Std. Err**.	***t***	***P***	**[95% Confidence Interval]**	
Group	1.21	1.16	1.04	0.302	−1.10	3.52
Sex	2.36	1.33	1.78	0.079	−0.28	5.00
PSS-baseline	0.42	0.12	3.52	0.001^*^	0.18	0.66
logEPA-baseline	1.41	1.05	1.34	0.185	−0.69	3.51
_cons	5.58	2.72	2.05	0.043	0.17	10.98

Indicator variables coding: Group: 0 = placebo, 1 = active; Sex: 0 = male, 1 = female;

* *Denotes significance; logEPA-baseline is the log of the baseline EPA (% of total fatty acids). The log was taken to comply with ITT (analyses all cases) and model assumptions (linear relationship with outcome), as there were a few extreme high values for each group that created a strong skew for untransformed EPA, as % to total fatty acids; The values for PSS-post for the participants who withdrew (n = 15) were the last PSS score available bought forward to allow intention to treat analysis (see also Figure 3)*.

### Compliance

A measure of compliance was obtained for 77% of the sample. Of these, 73% were compliant at the 85% level or higher. There were no differences between the groups or genders for compliance.

### Cortisol:DHEA ratio

The cortisol:DHEA ratio post-intervention was not influenced by treatment group (Table 5). However, there were strong trends toward significant effects and of sex (*p* = 0.07) and age (*p* = 0.06) on the ratio of cortisol to DHEA. The model in Table 5 shows that this ratio increased as age increased, and also that the ratio is increased for females.

**Table 5:** Coefficient table for the effect of treatment group on Cortisol:DHEA ratio (*n* = 75).

**Cortisol:dhea post**	**Coef**.	**Std. Err**.	***t***	***p***	**[95% Confidence Interval]**	
Group	−1.51	2.31	−0.66	0.514	−6.11	3.09
Sex	4.79	2.55	1.87	0.065	−0.31	9.88
Age	0.24	0.12	1.94	0.056	−0.01	0.49
Cortisol:dhea pre	0.61	0.11	5.63	<0.001^**^	0.39	0.83
Constant	−7.17	5.73	−1.25	0.215	−18.60	4.26

Dummy variables coding: Group: 0 = placebo, 1 = active; Sex: 0 = male, 1 = female;

** *Denotes significance at the p = 0.01 level*.

### Cytokines

There were no significant treatment effects on any of the cytokines or CRP. However, the PSS at time 2 was a significant predictor of both IL-6 (beta = 0.04, *t* = 2.75, *p* = 0.01) and CRP (beta = 0.03, *t* = 2.04, *p* = 0.05) controlling for the respective baseline measures. PSS at time 2 was also an inverse predictor of IL-10, after controlling for baseline IL-10, although not significantly (beta = −0.03, *t* = −1.32, *p* = 0.19).

### DASS

The group means for the DASS subscales are given in Table 3. The DASS subscales correlated with each other and the other stress measures, the stress subscale is shown in Table 6. There were no significant treatment effects on any of the DASS subscales.

**Table 6:** Correlation matrix for the various stress measures and IL-6 at time 2.

	**IL-6**	**PSS**	**DASS-Stress**	**OSI-Strain**
PSS	0.252			
*p*-value	0.031^*^			
*n*	73	75		
DASS-Stress	0.372	0.457		
*p*-value	0.004^**^	<0.001^**^		
*n*	57	59	59	
OSI-Strain	0.246	0.734	0.726	
*p*-value	0.068	<0.001^**^	<0.001^**^	
*n*	56	58	58	58
OSI-Resources	−0.009	−0.405	−0.203	−0.450
*p*-value	0.945	0.002^**^	0.123	<0.001^**^
*n*	57	59	59	58

* p is significant at the 0.05 level;

** *p is significant at the 0.01 level; IL-6 is interleukin-6; DASS-Stress is the stress subscale of the Depression and Anxiety Scale; OSI-R Occupational Stress Inventory-Revised; OSI-Strain is the Personal Strain Questionnaire (PSQ) subscale of the OSI-R; OSI-Resources is the Personal Resources Questionnaire (PRQ) subscale of the OSI-R*.

### OSI (occupational stress inventory)

The group means for the OSI-R subscales are given in Table 3. The OSI-R subscales correlated with each other and the other stress measures, shown in Table 6. There was no effect for group on the OSI subscales; the Occupational Roles Questionnaire (ORQ), the Personal Strain Questionnaire (PSQ) nor the Personal Resources Questionnaire (PRQ).

### Burnout

There was no difference between the groups at baseline for burnout, thus it was not included in the modeling as a covariate (Table 3).

### COPE denial

Group means for COPE Denial, COPE Active and COPE Disengagement are included in Table 3. In a general linear model of each of the 15 subscales of the COPE Inventory, only one subscale had significant effects for treatment group after baseline levels were taken into account. This was for the subscale, Denial. This subscale contained four items that asked questions such as: “I pretend that it hasn't really happened.” Sex was not a significant covariate in this model, so was not included. The sign of the coefficient is negative, indicating that scores on denial are lower for the active (EPA) group [indicator coding for group was 0 = placebo, 1 = active; *F*_(3, 56)_ = 16.94, beta = −1.17, *t* = −2.58, *p* = 0.013, 95% CI: −2.08, −0.26].

At post-intervention, Denial was correlated with IL6 (0.21, *p* = 0.116) and negatively correlated with EPA (*r* = −0.18, *p* = 0.177), although neither correlations were statistically significant in this sample size (*n* = 58). Denial was significantly correlated with the PSS (*r* = 0.38, *p* = 0.003), DASS Stress (*r* = 0.29, *p* = 0.027), DASS Anxiety (0.34, *p* = 0.008), DASS Depression (*r* = 0.46, *p* = 0.0003), OSI Psychological Strain (*r* = 0.30, *p* = 0.021), OSI Interpersonal Strain (*r* = 0.33, *p* = 0.012), OSI Physical Strain (0.44, *p* = 0.0005). It was not related to OSI Vocational Strain (0.044, *p* = 0.74).

### Blinding

To assess the success of the blinding, each participant was asked at the end of the study to indicate the supplement they believed they were taking during the trial. The available options were the active and placebo (fish oil and olive oil, respectively) and three other options (corn oil, linseed oil, or other oil). Interestingly, almost half of the fish oil group (18/37, 47%) correctly identified fish oil, while almost one-third of the placebo group incorrectly nominated fish oil (12/38, 32%) as the group to which they had been allocated. Conversely, only 4 (11%) from the placebo group correctly identified olive oil as the agent, where 3 (8%) from the fish oil group incorrectly identified olive oil.

## Discussion

This study found that there was no significant treatment effect for EPA on the Perceived Stress Scale (PSS-10) scores after 12 weeks of dietary supplementation in people with chronic work stress. ITT analysis was used, which included all participants who started the study. The 15 participants who withdrew were included by bringing their last PSS score forward. In some cases, the baseline score was used as the outcome score, potentially underestimating the true differences. However, in a sensitivity analysis, the effect of treatment group was similarly non-significant when only those who completed the study were included, after controlling for baseline stress and EPA and sex. Baseline EPA was included in the modeling as it was specified from the outset to be highly variable in the population; and indeed this variability was found in this sample, particularly for the active group.

The objective of this modeling was to assess the impact of treatment group on PSS scores after adjustment for baseline scores and significant covariates. The result was that the hypothesis that there would be a significant difference between the treatment groups at completion was not supported. However, the final model only accounted for approximately one-quarter (24%) of the variance in the outcome, perceived stress. This strongly suggests that there may be other significant factors that are omitted from this modeling. Hence, this result should be interpreted within the context of hypothesis testing and not as predictive model for stress in the population. Further exploratory modeling may explain more of the variance in the outcome to give a more complete understanding of the factors that influence stress outcomes, including a potential role for essential fatty acids, beyond the constraints of an ITT between-group difference hypothesis test.

Both groups experienced reductions in stress over time. This had been anticipated due to the regression-to-the-mean effect. The study recruitment had called for people with high work stress that had been ongoing for at least 3 months, so the scores were likely to fall. In our previous pilot study, we had shown a trend toward an *enhanced* reduction in stress in an active group taking a DHA-rich fish oil supplement (Bradbury et al., 2004). By conducting an *a priori* power analysis for the current trial, based the pilot study, the current trial was adequately powered to assess whether an EPA-rich fish oil supplement could significantly reduce stress over time in a sample from the population of people with chronic work stress.

There were some interesting sex differences on the stress outcomes in the current study. It was concerning that the random allocation to groups resulted in a lower proportion of males in the active fish oil group, while the ratio of males to females in the placebo group was relatively balanced. Males had significantly lower unadjusted stress scores than females at both time points. However, after adjusting for baseline PSS and baseline EPA levels, the effects of sex on the PSS scores at post-intervention were non-significant. Nevertheless sex was included as a covariate in all modeling to control statistically for the post-randomization imbalance between the groups. Experimental control through stratified allocation to larger groups should be considered in future studies using stress and coping as outcomes. Similarly, the pooling of data from multiple trials into a meta-analysis may help to control for the imbalance in the current study.

Another source of imbalance in the data was observed in the testing for agent identification at the end of the trial. Almost half (47%) of the fish oil group correctly identified fish oil, but only 11% of the placebo group correctly identified olive oil. Indeed, almost one-third (32%) of the placebo group believed themselves to have been allocated to the active fish oil group. This belief of by a substantial cohort of the control group has the potential to enhance the so-called “placebo effect,” which could under-estimate a true between-group difference, should one exist. This is a rigorous aspect of the experimental design which errs on the conservative side, giving rise to a preference for a type II error (failure to reject the null hypothesis) over a type I error (wrongly rejecting the null hypothesis). There had been 5% fish oil added to the placebo supplements to aid the blinding, which clearly worked as a successful masking strategy.

There were no effects for treatment group across any of the stress measures included as secondary outcomes in this study. While there was one significant effect found for EPA among the coping measures (the Denial subscale of the COPE inventory), it must be emphasized that this study was not designed to test conclusively for secondary outcomes. The significance level was not adjusted for multiple tests and there had been no *a priori* power analysis for secondary outcomes, so the findings on secondary outcomes must be interpreted as exploratory. As such, a range of secondary outcomes were included to enable such exploratory analyses for potential mechanisms and to suggest areas for future research.

Denial is considered a maladaptive coping mechanism, along with others such as disengagement. High levels of denial have been shown to predict worse outcomes for anxiety after cognitive behavior therapy (Oei et al., 2013). The current study finding that the EPA group had lower levels of denial than the placebo control group is consistent with modeling studies demonstrating an effect for omega-3 fatty acids on coping and resilience outcomes (Bradbury et al., 2012; Yoshikawa et al., 2015). While modeling was conducted with cross sectional data, and was therefore not designed to demonstrate causality, the current finding contributes to the mounting body of evidence suggesting a potential adaptogenic role for dietary omega-3 fatty acids.

There has been much interest in the inflammatory impact of stress in mental health disorders, and the potential of the omega-3 fatty acids to ameliorate this (McNamara, 2015). The current study found that interleukin 6 (IL-6) was correlated with all three stress measures (PSS, DASS-Stress, OSI-Strain). However, there was no direct correlation between the omega-3 fatty acids and stress or inflammatory measures. This finding is not consistent with Maes et al. (2000) who demonstrated that a membrane omega-3 fatty acids at baseline predicted proinflammatory cytokine changes during exam stress. It could be argued, however, that exam stress is a different form of stress, perhaps more acute and self-limiting, and not consistent with the type of chronic stress one encounters in the work place.

Exposure to ongoing work pressures could result in physiological adaptations; perhaps proinflammatory mediators, while initially raised, had subsided over time. Perhaps there was no correlation between the omega-3 fatty acids and the proinflammatory cytokines in the current study because the cytokines and C-reactive protein were not significantly elevated. A systematic review (Johnson et al., 2013) of work stress and C-reactive protein (CRP) reported on six studies that had examined the relationship, but only half found a significant relationship between work stress and CRP and the other half did not. Many of those analyses were subgroup analyses, secondary to the purpose of data collection. One of which used data from the Whitehall study, the large cohort of civil servants in the UK, which found the correlation was not significant.

By design, the chosen study intervention was high in EPA (83.3%) but low in DHA (16.6%). The decision to test for one and not a combination was influenced by an aim to contribute toward the debate as to which nutrient is most effective for various clinical populations. EPA was chosen in this study because it is a precursor to the family of eicosanoids that are antagonistic to families of potent proinflammatory mediators produced from arachidonic acid. As such, three proinflammatory cytokines were included as secondary outcomes to explore the nature and extent that EPA exerts an anti-inflammatory impact during stress, and thus provide preliminary evidence of the mechanism of effect.

There are three major limitations associated with this rationale that may have influenced the outcomes. Firstly, DHA has also recently been shown to be a potent anti-inflammatory precursor and may have more widespread neuroprotective roles than EPA (Sinclair et al., 2007; Bradbury, 2011). Recently, DHA and EPA have, respectively, been found to be precursors for families of molecules, called resolvins, that actively help to resolve inflammation; DHA for D-series and EPA for E-series (Qu et al., 2015). In addition, DHA is precursor for protectin (Protectin D1) and maresins. The latter are synthesized from DHA in macrophages to help regulate inflammation (Serhan et al., 2015). The discovery of these potent pro-resolving compounds implies a direct, active and localized role for DHA in tissue homeostasis.

Further, protectin D1 is also known as neuroprotectin D1 (NPD1) as its effects are largely within the brain. For instance, DHA supplementation was shown to reverse the age-related declines in adenosine triphosphate (ATP) production in isolated mouse brain tissue, an action which corresponded with increased NPD1 levels (Afshordel et al., 2015). In cytokine-stressed (i.e., LPS-stimulated) human brain cells, NPD1 was found to reduce amyloid-beta secretion, hence showing promise to protect against Alzheimer-linked degeneration (Lukiw et al., 2005; Palacios-Pelaez et al., 2010). Thus, in addition to its anti-inflammatory and pro-resolving roles, DHA is also increasingly recognized for its promising neuroprotective properties.

Given that psychological stress is largely regulated in the brain, perhaps the low DHA in the current study was a design limitation. Indeed, in mice subjected to stressful tasks, such as forced swimming, a DHA-rich diet helped to significantly reduce multiple stress hormones, including ACTH (by 25%) and corticosterone (by 20%; Jiang et al., 2015). Further, DHA was shown to be more effective than EPA at reducing IL-1β and IL-6, while EPA was more potent at reducing TNF-α (Mullen et al., 2010).

Secondly, the dosage of both EPA and DHA are low compared to the equivalent used in animal studies and may have been too low to have exerted a pronounced effect. We used 2,200 mg EPA + 44 mg DHA per day for 12 weeks, which is a ratio of 83.3% EPA to 16.6% DHA. This is consistent with another recent RCT in 261 healthy adults that found that 1,000 mg EPA + 400 mg DHA per day for 18 weeks did increase membrane levels by 64% but did not suppresses *ex vivo* production of C-reactive protein or proinflammatory cytokines (Muldoon et al., 2016). The authors similarly speculated that this may be due to low dosage or perhaps the activation of other anti-inflammatory pathways that were not included in the study.

Others have demonstrated that in *ex vivo* and *in vitro* studies, the concentration of LPS used to stimulate the proinflammatory cytokines influences the anti-inflammatory activity of omega-3 fatty acids; lower LPS concentrations were associated with a larger anti-inflammatory response by omega-3 fatty acids (Mullen et al., 2010). The current study used circulating levels of cytokines, which circumvents this particular methodological issue. However, capturing naturally occurring cytokines may also increase inter-individual variability in the data as many factors may influence naturally occurring proinflammatory cytokine levels, such as the nature, extent and timing of the exposure to the stressor.

The dosage of omega-3 fatty acids required to cause a clinical effect in humans is still largely uncertain. In rats, dosage may range from 0.4 to 1 g/kg/day of EPA + DHA (Pusceddu et al., 2015), which would correspond to a dosage range from 28 to 70 g/d in a 70 kg human. The NHMRC recommends an upper limit of 3,000 mg/d (Nutrient Reference Values for Australia New Zealand, 2005). However, in a large RCT (*n* = 302) of older adults in The Netherlands, two different doses were used (“high” dose: 1,093 mg EPA + 847 mg DHA, which corresponds to eating eight portions of fish per week; “low” dose: 226 mg EPA + 176 mg DHA, corresponding with one or two fish meals per week) were given for 26 weeks. There was no change in the primary outcome, Quality of Life (QOL) scores, for either dose. This may have been because the “high” dose is still too low to exert a clinical effect. While it may increase membrane levels of the fatty acids, it may be at a basic physiological level, to correct a widespread population dietary imbalance (Meyer, 2011), which may still be too low to be detected by clinical assessments.

It may also be that the combination of EPA and DHA is more effective than either fatty acid alone. An animal study that investigated EPA vs. DHA vs. the combination of EPA + DHA on *in vitro* production of proinflammatory cytokines cultured from rat monocytes found that the combination of EPA + DHA had the strongest effect, which they attributed to synergistic actions through multiple pathways (Zhang et al., 2015). In a study in humans, the combination of 2,710 mg EPA/day + 2,040 mg DHA/day for 4 weeks significantly reduced cigarette smoking and cravings in 48 smokers (Rabinovitz, 2014). The increase in DHA in that study meant that the participants were getting double the dose of n-3FAs as those in our study and the Dutch study. Perhaps future research needs to consider not only increasing but balancing the dosage of both EPA and DHA in supplementation to optimize both physiological and clinical effects.

Finally, the physiological state of psychological stress is not a stable condition; the organism moves from an acute/emergency phase of stress into later adaptive phases if exposure is prolonged. In this study, chronic work stress was used as a model of stress because it was thought to be relatively stable. While at least one meta-analysis found that levels of proinflammatory cytokines are increased during acute psychological stress (Steptoe et al., 2007), an earlier and broader meta-analysis of more than 300 original studies found that most arms of the immune system are suppressed during chronic stress (Segerstrom and Miller, 2004). It would seem that during chronic stress the immune system is already suppressed through other regulatory mechanisms, thus there is no requirement to further suppress it. Hence, a regulatory role of omega-3 fatty acids may be more complex than simply antagonistic effects toward proinflammatory cytokines. If an immune system is already chronically suppressed due to stress mechanisms, it would not seem logical or desirable to further suppress it; rather, it may be more desirable to stimulate it. If a cytokine response to stress is not already exaggerated, it may not be desirable for omega-3 fatty acids to blunt a healthy cellular response to a stressor. It would seem therefore that there are multiple mechanisms regulating the proinflammatory cytokines during chronic stress, and that the role of omega-3 fatty acids may be much more regulatory and subtle than this study was designed to capture.

To further investigate a role for omega-3 fatty acids in stress, particularly with regards to the inflammatory impact of stress, future studies could focus on the prevention of an exaggerated response to an acute stressor, rather than a reduction of chronic stress. Most of the early stress work was conducted in university environments in fit and healthy, young, male medical students using exam stress as a model for stress. However, the ability to control experimentally for many potential cofactors and thus the strong internal validity comes at a cost of poor external validity. The findings from young, healthy males in their prime may not be generalizable to a general and aging population. More pragmatic studies in stress in the general population are needed, so the difficulties of modeling chronic stress will need to be addressed.

Future studies should address the role of DHA in stress, with particular emphasis on coping mechanisms. Perhaps DHA or a better balance of EPA and DHA may yet prove to have more of an impact in stress, resilience and coping. A recent pilot study reports that 75% of psychiatric patients screened were found to have < 4% EPA + DHA as a proportion to total fatty acid levels in whole blood, compared with 25% of the general population in the U.S. (Messamore and McNamara, 2016). A study on a prison population in Australia found a median EPA + DHA of 4.7% as a proportion to total fatty acids, indicating that almost half of all the prisoners had very low cell membrane levels, which was correlated with increased aggression and attention deficit disorder (Meyer et al., 2015). Thus, the “omega index” (EPA + DHA) may be more important than either nutrient in isolation for obtaining clinically assessable effects. Further work is required to standardize the omega index (von Schacky, 2014).

Finally, stress is a multidimensional construct, and its representation as a single global factor does not capture the subtly differentiated relationships between the various stress factors, stress biomarkers and the fatty acids. The PSS has been found to have a three factor structure, with cytokines and fatty acids having differential relationships with the different factors (Bradbury et al., 2012; Bradbury, 2013). Future mediation modeling with this data may find more indirect relationships among the variables when stress is represented as a multidimensional construct.

## Conclusion

This study demonstrated that 2.2 g/day of an EPA-rich fish oil for 12 weeks did not reduce psychological stress in people with chronic work stress.

## Author contributions

JB, SM, and LB conceived and designed the RCT; BM, AS, and JP performed the blood analyses; JB conducted saliva analyses; JB and LB analyzed the data; CS and AS contributed strategic expert planning and advice; JB drafted the manuscript.

### Conflict of interest statement

The authors declare that the research was conducted in the absence of any commercial or financial relationships that could be construed as a potential conflict of interest. The sponsors had no role in the design of the study; in the collection, analyses, or interpretation of data; in the writing of the manuscript, and in the decision to publish the results.
